# Immune Response Activation and Hepatoprotective Activity of *Randia echinocarpa* Soluble Melanins in Murine Models

**DOI:** 10.1155/ijfo/5888390

**Published:** 2025-04-14

**Authors:** María del Rosario Gil-Avilés, Sylvia Paz Díaz-Camacho, Ulises Osuna-Martínez, Gabriela López-Angulo, Francisco Delgado-Vargas

**Affiliations:** ^1^Research Unit in Biomedical Biotechnology, Autonomous University of Occident, Culiacan, Sinaloa, Mexico; ^2^School of Chemical and Biological Sciences, Autonomous University of Sinaloa, Culiacan, Sinaloa, Mexico

**Keywords:** functional food, hepatoprotective, lymphocytes T *γδ* expression, plant melanins, plant pigments

## Abstract

This research demonstrates the in vivo immunomodulatory and hepatoprotective activities of the soluble melanins of the *Randia echinocarpa* fruit (PSM). The splenocyte cellular metabolic activity and lymphocyte T *γδ* expression in mesenteric lymph nodes (MLNs) and Peyer patches (PPs) were measured in the mice model. The PSM hepatoprotective activity was evaluated in the CCl_4_-induced acute hepatotoxic injury (AHTI) in the rat model. Compared with the controls, the PSM treatment induced higher splenocyte cellular metabolic activity (in vitro, 24.1%–57.25%; in vivo, 28.8%–47.7%), activation of lymphocytes T *γδ* in MLN but suppression in PP. Related to in vivo hepatoprotective activity, PSM treatment reduces CCl_4_-induced damage; animals showed lower levels of serum ALT (218.85–67.02 U/L) and ALP (453.37–355.47 U/L), higher levels of serum GSH (127.96–252.15 ng/mg of tissue), lower levels of hepatic MDA (10.25–7.85 nmol/mL), and less severe damage in the hepatic histopathology. These results suggest the nutraceutical and therapeutic potential of PSM.

## 1. Introduction

The *Randia echinocarpa* fruit, known as “papache” in Sinaloa, Mexico, has long been used in ethnopharmacology to treat diabetes, cancer, and malaria [1, 2]. This versatile fruit has also shown diverse biological activities, including antioxidant and antimutagenic properties [3]. A hexane fraction of the fruit exhibited high antimutagenic activity due to its *β*-sitosterol, linoleic acid, and palmitic acid content, suggesting its potential as an antitumoral and anticancer agent [4]. Furthermore, *R. echinocarpa* melanins have mitogenic properties on mice splenocytes and antioxidant activity against oxidative stress in *Saccharomyces cerevisiae* [5]. These diverse biological activities of the papache fruit awaken scientific interest and underscore its potential for further research and application.

Melanins are widely distributed in living organisms and serve various functions. They offer protection against UV radiation and oxidative stress, contribute to drug resistance in pathogens, provide insect protection against bacteria and viruses [6, 7], act as chelating agents of metals, and function as redox agents [8]. Additionally, melanins exhibit antioxidant properties [9–15] and possess immunomodulatory activities in various models [5, 16–18]. Melanins from various sources have also shown hepatoprotective activity, including from tea [19], *Lachnum* sp. [20], *Streptococcus parvus* [21], and *Inonotus obliquus* [22]. However, pure melanins are typically insoluble in water, which limits their physicochemical characterization and applications. Nevertheless, water-soluble forms of melanin can be found in natural sources, including papache fruit. Cuevas-Juárez et al. [9] demonstrated in vitro biological activities of papache soluble melanins (PSMs), such as *α*-glucosidase inhibition and antioxidant effects. Specifically, the PSM antioxidant activity evaluated by ABTS (719.5 *μ*mol TE/g) and FRAP (351.8 *μ*mol TE/g) was high, registering values similar to those of coffee beans [9]. Moreover, the structural characteristics of papache fruit melanins were previously reported [5, 9, 23]. Furthermore, PSM is safe in acute (LD_50_ > 5 g/kg bw) and subacute (500 mg/kg bw/day/30 days) toxicity assays in mice [23]. However, limited information is available on the in vivo biological activities of papache fruit extracts. Considering the range of ethnopharmacological uses of *Randia echinocarpa* fruit, a plant whose habitats are being destroyed, it is important to demonstrate its health-beneficial properties, particularly its hepatoprotective and immunomodulatory activities. Products with such properties are highly pertinent nowadays since humans are continuously exposed to hepatic toxic substances, and oxidative stress induces failure in the immune system. Therefore, the objectives of this research were to evaluate the immunomodulatory and hepatoprotective activities of PSMs.

## 2. Materials and Methods

### 2.1. Plant Material

The fruits of *R. echinocarpa* (Sessé et Mociño) were collected in Salvador Alvarado and Badiraguato, Sinaloa, Mexico, and transported to the School of Chemical and Biological Sciences, Autonomous University of Sinaloa (UAS). The plants were identified and authenticated with a voucher specimen (8040) deposited in the herbarium by Dr. Rito Vega-Aviña, School of Agronomy, UAS. The woody peel and the seeds of the fruits were removed; the edible pulp was then recovered, stored at −40°C, and freeze-dried to obtain a fruit powder that passed through a No. 30 mesh. The powder was stored at −20°C and protected from light until used.

#### 2.1.1. Extraction and Purification of PSMs

PSMs were obtained as described by Cuevas-Juárez et al. [9]. Briefly, the raw water-soluble melanins were obtained by mixing and agitating 5 g of *R. echinocarpa* powder and 100 mL of deionized water in an amber flask for 30 min at boiling temperature. The suspension was centrifuged at 20,000 g/20°C for 15 min; the supernatant was recovered, stored at −70°C, and freeze-dried. The freeze-dried raw-soluble melanin (0.5 g) was suspended in 10 mL of deionized water and dialyzed using a prehydrated nominal 12 kDa cellulose membrane (Sigma-Aldrich, Seelze, Germany) in a flask containing 300 mL of deionized water. The dialysis process was carried out for 4 days at 5°C, with the water in the flask being exchanged twice a day. The dialysis retentate was then recovered, stored at −70°C, and freeze-dried to obtain the PSMs [9].

### 2.2. Animals

BALB/c mice (Bioinvert, S.A. de C.V., Mexico) were used for the immunomodulatory studies. The in vivo antioxidant activity was evaluated in fifty 6-week-old male Wistar rats provided by the Animal Research Vivarium of Coapa-CINVESTAV Unit in Mexico City. The animals were maintained at 24 ± 2°C (50% humidity/12 h light–dark cycles) and provided water and feed (Nutricubos, Nutrimentos Purina S.A. de C.V., Mexico) ad libitum. All animal procedures and experiments were conducted with the approval of the Ethics Committee of the School of Veterinary of the Autonomous University of Sinaloa and according to the Mexican Official Norm NOM-062-ZOO-1999 and the guide for the care and use of laboratory animals [24].

### 2.3. Immunomodulatory Activity of the PSMs

#### 2.3.1. In Vitro and In Vivo BALB/c Mice Splenocyte Cellular Metabolic Activity

Mice splenocyte cellular metabolic activity was measured as reported by Lin et al. [25] using the 3-(4,5-dimethyl-2-thiazolyl)-2,5-diphenyl-2H-tetrazolium bromide (MTT) assay, with some modifications. Two BALB/c mice (6-week-old) were anesthetized with pentobarbital, sacrificed, and disinfected with 10% (v/v) benzal solution, and the spleen was aseptically recovered. The spleen was homogenized in the DMEM medium using a sterile syringe plunger in a Petri dish. The cell suspension was centrifuged (200 g × 5 min) (EBA 20 Hettich Zentrifugen, Tuttlingen, Germany), and the supernatant was discarded. The pellet was mixed with 10 mL of lysis buffer (pH 7.2–7.4) and incubated for 10 min to eliminate erythrocytes. After centrifugation (200 g × 5 min), the splenocytes were recovered in the pellet. The splenocytes were suspended in DMEM complemented with 10% v/v fetal bovine serum and mixed with 1% v/v antibiotic and antimycotic (Sigma-Aldrich, St. Louis, MO, United States), adjusting the cell count to 1 × 10^6^ cells/mL. In a 96-flat bottom microwell plate, 200 *μ*L of the splenocyte suspension was mixed with pure DMEM (control group) or added with 50 *μ*L of LPS (10 *μ*g/mL) or different PSM concentrations (1, 10, 50, 100, and 200 *μ*g/mL) [25]. The LPS and PSM were prepared as solutions in DMEM medium. The plate was incubated at 37°C, 5% CO_2_, and 95% humidity for 24 h (Walter Jacket Automatic CO_2_ Incubator, NuAire, Plymouth, MA, United States). Each well was added with 10 *μ*L of 5 mg/mL MTT in PBS, and the tetrazolium salts formed by the viable cells were dissolved by agitation in 100 *μ*L of dimethyl sulfoxide (DMSO). The absorbance at 540 nm was measured using a microplate reader (Multiskan Bichromatic, Fisher Scientific, Waltham, MA, USA), and cellular metabolic activity was expressed as a percentage.

For the in vivo splenocyte proliferation assay, 18 BALB/c male mice (6-week-old) were divided into three groups (*n* = 6) and treated for 14 days as follows: PSM group treated with PSM (10, 50, and 100 mg/kg bw), control group treated with water (10 mL/kg bw), and positive control treated with LPS (10 *μ*g/mouse) (Sigma-Aldrich). The animals were maintained at 24 ± 1°C and provided ad libitum feed and water. After the treatment period, the mice were anesthetized and sacrificed. The spleen was recovered in PBS, added with 1% FBS, sodium azide, and 1 mM EDTA, and mechanically disaggregated using two frosted slides. The cell suspension was passed through 70 *μ*m mesh, mixed with 300 *μ*L PBS, and centrifuged (1000 × g/10 min/4°C). The pellet was suspended in PBS and mixed with 0.4% trypan blue (1:1 v/v) in a Neubauer chamber to count the number of viable cells.

#### 2.3.2. In Vivo Effect of the PSM on Three Lymphocyte Cell Lines in Mesenteric Lymph Nodes (MLNs) and Peyer's Patches (PPs): B, T *αβ*, and T *γδ*

This assay was carried out as Ramiro-Puig et al. [26] described it with some modifications. Briefly, ten 6-week-old BALB/c male mice with an average weight of 20–25 g were divided into two groups (*n* = 5). One group was treated by intragastric administration with PSM suspended in water (100 mg/kg bw) for 4 days, while the other group received water only (10 mL/kg bw). After treatment, the mice were anesthetized, sacrificed, disinfected with 70% ethanol, and dissected to recover the MLNs and PPs. These tissues were placed in PBS (containing 1% FBS, 0.01% sodium azide, and 1 mM EDTA) and mechanically disaggregated using two frosted slides. The resulting suspension was filtered through a 70 *μ*m mesh, mixed with 300 *μ*L PBS, and centrifuged (1000 × g/10 min/4°C). The recovered pellet was mixed with 2% universal blocker (1×) in casein suspension. The mixture was incubated at 4°C for 15 min in the dark, and the suspension was adjusted to 1 × 10^6^ cells/tube and washed with 300 *μ*L PBS.

The cell suspensions were stained with the following primary antimice antibodies (BD, PharMingen, United States): anti-CD45R (B lymphocytes), anti-CD3 (T *αβ* lymphocytes), and anti-*γδ* (T *γδ* lymphocytes) for cellular immune typification and quantitation. Additionally, anti-CD69-PerCp5.5 was used to determine the activation state of the three cell lines. The antibody selection was based on lymphocyte immunophenotyping: B lymphocytes (CD45R^+^), T *αβ* lymphocytes (CD3^+^*γδ*^−^), and T *γδ* lymphocytes (CD3^+^*γδ*^+^). The activation state of these cells is associated with the CD69^+^ marker. Therefore, the following antibody mixtures were used: B lymphocytes (CD45R-FITC and CD69-PerCp5.5) and T lymphocytes (CD3-FITC, *γδ*-PE, and CD69-PerCp5.5). The cell suspension (1 × 10^6^ cells/tube) with the corresponding antibody mixture was incubated at 4°C for 25 min in the dark, washed with 300 *μ*L PBS, and centrifuged at 1000 × g for 5 min. The samples were then fixed with 400 *μ*L of 1% *p*-formaldehyde (PFA) and analyzed with a flow cytometer (Beckman Coulter, Indianapolis, IN, United States) [26] at the Center of Research and Advanced Studies (CINVESTAV) of the National Polytechnic Institute (IPN) in Mexico City, Mexico. The data were analyzed with the FlowJo V10 software (BD Biosciences, Franklin Lakes, NJ, United States) to obtain the cellular percentages.

### 2.4. Hepatoprotective Activity of the PSM

#### 2.4.1. CCl_4_-Induced Acute Hepatotoxic Injury (AHTI) Model

The CCl_4_-induced AHTI model was carried out as described by [27]. The dose of PSM in this evaluation (33 mg/kg bw i.p.) was chosen based on previous reports involving similar melanins, such as those from apricot kernel [28] and tea [19]. The doses were adjusted before each administration to account for any changes in body weight of rats over the entire treatment period for each group. Briefly, thirty-six 4-week-old Wistar rats (120 g ± 20 g bw) were divided into six groups (*n* = 6): (1) untreated control group, (2) vehicle-treated group receiving saline solution (0.09% w/v, 10 mL/kg bw, at Days 1, 3, and 5), (3) PSM-treated group (33 mg/kg bw i.p., at Days 1, 3, and 5), (4) group with CCl_4_-induced AHTI (2 mL CCl_4_/kg bw i.p., at Day 5), (5) group with AHTI pretreated with silymarin (50 mg/kg bw p.o., at Days 1, 3, and 5), (6) and group with AHTI pretreated with PSM (33 mg/kg bw i.p., at Days 1, 3, and 5). During this time, rats were weighed on Days 1, 3, and 5.

On the sixth day, 24 h after the treatment with CCl_4_, rats were anesthetized with phenobarbital. Blood extracted by cardiac puncture was centrifuged at 1600 × g for 10 min, and obtained serum was stored at −70°C until the biochemical assays. Rats were euthanized, and the liver was recovered, weighed, and stored in 10% neutral buffered formalin for histopathological studies.

#### 2.4.2. Serum Enzyme Determinations

Serum alkaline phosphatase (ALP), aspartate aminotransferase (AST), and alanine aminotransferase (ALT) activities were measured using Weiner Lab kits following the manufacturer's instructions. Briefly, the ALP activity was determined by mixing 3 *μ*L of serum with the kit reagents. The mixture was then incubated at 37°C for 3 min, and absorbance at 405 nm was measured using a microplate reader, ELIREAD (KontroLab, Naples, Italy). ALP values were expressed as international units per liter (U/L). For AST and ALT determinations, 40 *μ*L of serum was mixed with the kit reagents. The mixture was incubated at 37°C for 3 min, and the absorbance at 340 nm was measured using a spectrophotometer, Spectronic Genesys 5 (Spectronic Instruments, Melville, NY, United States). ALT and AST values were expressed as U/L.

#### 2.4.3. Determination of Oxidative Stress Markers: Glutathione (GSH) and Malondialdehyde (MDA)

GSH was quantified using the Biovision GSH colorimetric detection kit (Catalog no. K261-100). A liver sample (100 mg) and 400 *μ*L of buffer were coldly homogenized in an Eppendorf tube. The mixture was then added with 100 *μ*L of 5% sulfosalicylic acid and sonicated for 1 min. Samples were centrifuged at 8000 × g for 10 min at room temperature (25°C), and the supernatant was recovered. Next, an aliquot of the supernatant (20 *μ*L) or standard solution was mixed with NADPH and incubated for 7.5 min at room temperature and darkness. Then, 20 *μ*L of GSH substrate solution was added, and samples were incubated for 10 min at room temperature. The absorbance of the samples at 405 nm was measured using a microplate reader, ELIREAD (KontroLab). GSH concentration was determined with a calibration curve and reported as micromolar of GSH per milligram of liver tissue.

MDA concentration was determined using the Biovision lipid peroxidation colorimetric/fluorometric assay kit (catalog no. K739-100). Serum (10 *μ*L), 250 *μ*L of 42 mM H_2_SO_4_, and 62.5 *μ*L of phosphotungstic acid were mixed, left at room temperature for 5 min, and then centrifuged (13,000 × g/3 min/room temperature). The pellet was recovered, suspended in 49 *μ*L of H_2_Odd, added with 1 *μ*L of BHT, and the mixture was made up to 100 *μ*L with distilled water. A calibration curve was prepared with different volumes (1, 2, 3, 4, and 5 *μ*L) of 2 mM MDA. Next, samples or the standard solution was added with 300 *μ*L of TBA and incubated at 95°C for 60 min. Then, the tubes were added with 150 *μ*L of *n*-butanol and 100 *μ*L of 5 M NaCl, mixed, and centrifuged (16,000 × g/3 min/room temperature). The *n*-butanol phase was recovered and concentrated at 55°C, and the residue was suspended in 100 *μ*L of H_2_Odd and mixed. Aliquots of each tube (75 *μ*L) were placed in the microplate, and the absorbance at 532 nm was measured in dark conditions using the microplate reader, ELIREAD (KontroLab). Results were reported as nanomole of MDA per milliliter of serum.

#### 2.4.4. Histopathological Analysis of Liver Tissue

In the assay, the methodology of Parfenov et al. [22] was followed. Briefly, liver samples were fixed with a formalin buffer solution containing 100 mL/L of 10% formaldehyde (J.T.Baker), 4 g/L of NaH_2_PO_4_ (Vetec), and 6.5 g/L of Na_2_HPO_4_ (Fermont) at a pH of 7.4. The tissue samples were cut into 0.5 cm × 2.0 cm sections, dehydrated, and embedded in paraffin (Leica Paraplast). Subsequently, 5–7-*μ*m-thick samples were obtained using a Leica RM2125 RTS microtome, placed on two slides, stained with hematoxylin and eosin (H&E), and analyzed at 10× and 40×. This procedure was performed by two researcher pathologists blinded with the analysis of 30 fields per sample looking for necrosis, apoptosis, steatosis, ballooning degenerations, inflammation, and other parameters using an optical microscope, Primo Star (Carl Zeiss, Oberkochen, Germany). The degree of liver steatosis was analyzed according to the scoring system of the Nonalcoholic Steatohepatitis Clinical Research Network (NASH CRN) [29].

### 2.5. Statistical Analysis

Data was analyzed by one-way ANOVA, and statistical differences among means were established by the Fisher test (*p* < 0.05). The employed software was GraphPad Prism 6.0 (GraphPad Software, LLC Company, Boston, MA, United States).

## 3. Results

### 3.1. Immunomodulatory Activity of the PSMs

#### 3.1.1. Increased Cellular Metabolic Activity of Splenocytes

**Figure 1:**
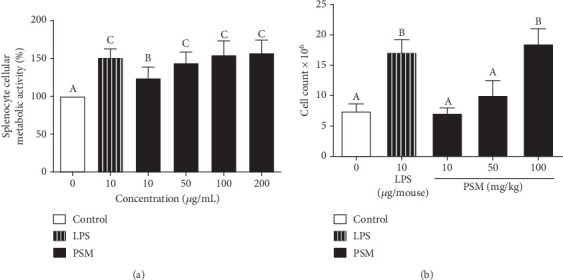
Effect of papache soluble melanins (PSM) on (a) splenocyte cellular metabolic activity in vitro and (b) splenocyte proliferation in vivo. LPS: lipopolysaccharide. Each bar represents the mean ± standard deviation (*n* = 3). Data were analyzed using a one-way ANOVA and Fisher multiple comparison test. Different letters on the bars indicate significant differences between treatments (*p* < 0.05).

The PSM showed in vitro immunomodulatory activity by increasing the cellular metabolic activity of mouse splenocytes by 24.1%–57.25%. The cellular metabolic activity tended to increase with a dose range of 10–200 mg/mL and was significantly different from the negative control mice. Furthermore, the splenocyte cellular metabolic activity values, starting from 50 *μ*g/mL PSM, were similar to or higher than those obtained with the mitogen LPS (51%) (*p* > 0.05) (Figure 1a).

For the in vivo splenocyte proliferation assay, mice treated with PSM exhibited a dose–response effect (10–100 mg/kg bw). In this regard, the proliferation in mice treated with 100 mg/kg PSM (47.7%) was higher than that of control mice (28.8%) (*p* < 0.05) and similar to that of LPS-treated mice (Figure 1b).

#### 3.1.2. Effect of PSM Intragastric Administration on Lymphocyte Populations in MLNs and PPs in Mice

**Figure 2:**
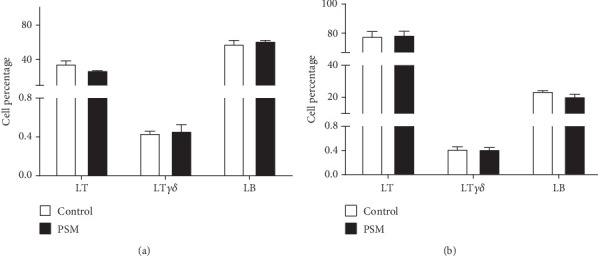
Effect of oral administration of papache soluble melanins (PSMs) on cell percentage of lymphocytes in (a) Peyer's patches (PPs) and (b) mesenteric lymph nodes (MLNs) of BALB/c mice: T (LT), T *γδ* (LT*γδ*), and B (LB) lymphocytes. The results are expressed as the mean ± standard deviation (*n* = 5). Data were analyzed using a one-way ANOVA and Fisher multiple comparison test. The values for the control and PSM-treated mice were similar for each parameter (*p* ≥ 0.05). Mice treatments for 4 consecutive days: control, 100 mL of injectable water; PSM, 100 mg of PSM/kg bw.

**Figure 3:**
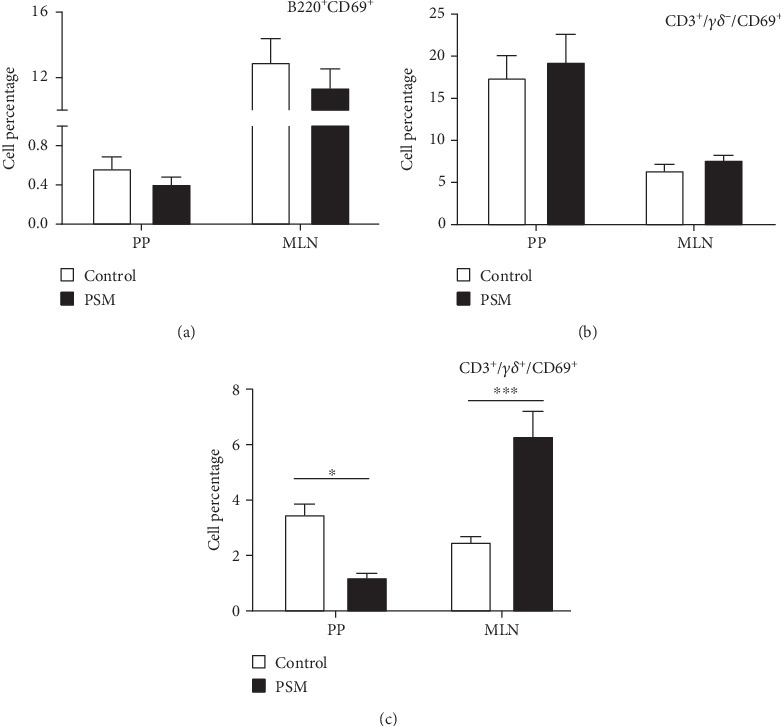
Effect of oral administration of papache soluble melanins (PSMs) in the activation of lymphocytes (B, T *αβ*, and T *γδ*) in mesenteric lymph nodes (MLNs) and Peyer's patches (PPs) of BALB/c mice. The results are expressed as the mean ± standard deviation (*n* = 5). Data were analyzed using a one-way ANOVA and Fisher multiple comparison test. Panels show the cellular percentages of lymphocytes: (a) B, B220^+^CD69^+^; (b) T *αβ*, CD3^+^/*γδ*^−^/CD69^+^; (c) T *γδ*, CD3^+^/*γδ*^+^/CD69^+^. Statistical differences: ⁣^∗^*p* < 0.01 and ⁣^∗∗∗^*p* < 0.001. Mice treatments for 4 consecutive days: control, 100 mL of injectable water; PSM, 100 mg of PSM/kg bw/mouse.

In mice treated with PSM (100 mg/kg/4 days), the cellular percentage of T *αβ*, T *γδ*, and B lymphocytes in MLN and PP was not affected (Figure 2). Similarly, PSM did not activate the B (CD19^+^ and CD69^+^) and T *αβ* (CD3^+^/*γδ*^−^/CD69^+^) lymphocytes (Figure 3a,b), but the T *γδ* (CD3^+^/*γδ*^+^/CD69^+^) lymphocytes were activated in the MLN and suppressed in PP (Figure 3c).

### 3.2. Hepatoprotective Activity of the PSMs

#### 3.2.1. Serum Enzyme Levels in Treated Rats

The levels of serum enzymes (ALT, AST, and ALP) in rats from the negative control, vehicle, and PSM groups without hepatic damage were similar (*p* > 0.05). However, higher levels (*p* < 0.05) were observed in rats with CCl_4_-induced AHTI (Table 1). Silymarin and PSM exhibited a hepatoprotective effect in groups of rats with AHTI. Compared with the enzyme levels in rats of the hepatotoxic group (AHTI), lower ALT levels and similar AST levels were recorded in rats pretreated with silymarin or PSM (Table 1). The hepatoprotective effect of the PSM was also observed in the ALP levels, as the PSM-treated rats exhibited lower values compared to rats with AHTI (Table 1).

#### 3.2.2. Effect of PSMs Administered to Mice on the Levels of GSH in Hepatic Tissue and MDA in Serum

Rats pretreated with silymarin or PSM exhibited a reduction in CCl_4_-induced oxidative stress, as indicated by higher levels of hepatic GSH compared to rats treated only with CCl_4_ (group AHTI). However, the AHTI group of rats, without pretreatment, did not show reduced GSH levels compared to animals in the negative control, vehicle, and PSM groups without damage (Table 1).

The analysis of the rat MDA levels in serum demonstrated lower values in rats pretreated with silymarin and PSM compared to the AHTI group of animals (*p* = 0.0001). The higher MDA levels in the AHTI group indicated that these rats were exposed to higher oxidative stress than those in the control groups (negative, vehicle, and PSM without AHTI) (Table 1).

#### 3.2.3. Effect of PSM Treatment on Rat Hepatic Morphology

**Figure 4:**
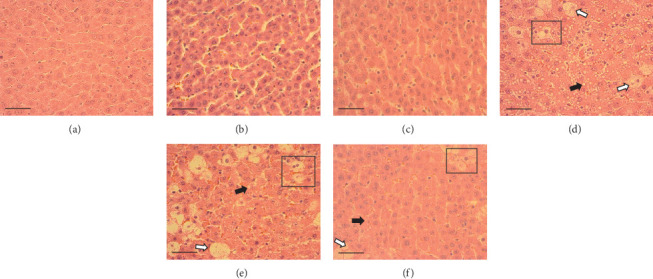
Effect of papache soluble melanin (PSM) treatment on liver parenchymal morphology of Wistar rats with acute hepatotoxic injury (AHTI). Hepatic tissue was treated with hematoxylin and eosin stain and observed at 40× magnification. Rat treatments: (a) control group; (b) vehicle group, saline solution (0.09% w/v); (c) PSM group (PSM 33 mg/kg bw via i.p.); (d) AHTI group (CCl_4_ 2 mL/kg bw via i.p.); (e) silymarin group + AHTI (silymarin 50 mg/kg bw via p.o. and CCl_4_ 2 mL/kg bw via i.p.); and (f) PSM group + AHTI (PSM 33 mg/kg bw via i.p. and CCl_4_ 2 mL/kg bw via i.p.). Rectangles = ballooning cells, white arrows = cells with macrovesicular steatosis, black arrows = microvesicular steatosis. Scale bar = 50 *μ*m.

**Figure 5:**
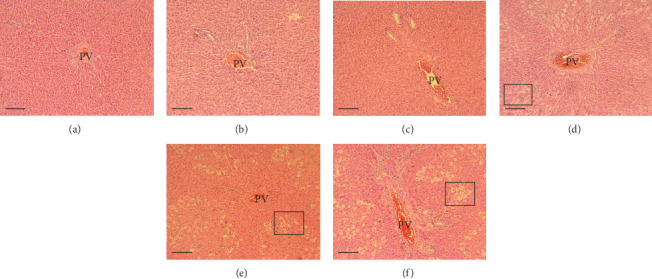
Effect of papache soluble melanin (PSM) treatment on liver morphology of the portal structure of Wistar rats with acute hepatotoxic injury (AHTI). Liver tissues were treated with hematoxylin and eosin stain and viewed at 10× magnification. Rat treatments: (a) control group; (b) vehicle group, saline solution (0.09% w/v); (c) PSM group (PSM 33 mg/kg bw via i.p.); (d) AHTI group (CCl_4_ 2 mL/kg bw via i.p.); (e) silymarin group + AHTI (silymarin 50 mg/kg bw via p.o. and CCl_4_ 2 mL/kg bw via i.p.); and (f) PSM group + AHTI (PSM 33 mg/kg bw via i.p. and CCl_4_ 2 mL/kg bw via i.p.). Rectangles = ballooning cells, PV = portal vein. Scale bar = 200 *μ*m.

**Figure 6:**
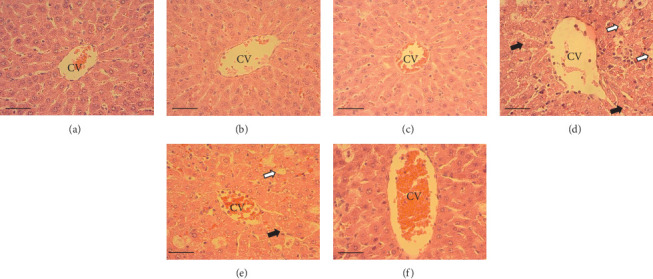
Effect of papache soluble melanin (PSM) treatment on hepatic morphology of the central vein of Wistar rats with acute hepatotoxic injury (AHTI). Hepatic tissue stained with hematoxylin and eosin stain, observed at 40× magnification. Rat treatments: (a) control group; (b) vehicle group, saline solution (0.09% w/v); (c) PSM group (PSM 33 mg/kg bw via i.p.); (d) AHTI group (CCl_4_ 2 mL/kg bw via i.p.); (e) silymarin group + AHTI (silymarin 50 mg/kg bw via p.o. and CCl_4_ 2 mL/kg bw via i.p.); and (f) PSM group + AHTI (PSM 33 mg/kg bw via i.p. and CCl_4_ 2 mL/kg bw via i.p.). White arrows = ballooning cells, black arrows = microvesicular steatosis, CV = central vein. Scale bar = 50 *μ*m.

The hepatic parenchyma of rats in the negative control (Figures 4a, 5a, and 6a), vehicle (Figures 4b, 5b, and 6b), and PSM (Figures 4c, 5c, and 6c) groups exhibited a normal architecture. The parenchyma consisted of hepatocyte cords composed of cells with the typical polyhedral form (did not show inflammatory infiltrates) (Figures 4a, 5b, and 6c), porta structure (Figures 5a, 5b, and 5c), and central vein (Figures 6a, 6b, and 6c). No necrotic or apoptotic cells were observed, and the level of steatosis according to the NASH CRN score was categorized as zero [30].

In contrast, the AHTI group (Figures 4d, 5d, and 6d) displayed a severe damage characterized by necrosis, inflammation, a high number of ballooned cells, and macrovesicular–microvesicular steatosis at Level 2 in Zones 1 (periportal), 2 (central), and 3 (perivenular) of the analyzed hepatic tissues.

The group treated with silymarin + CCl_4_ (Figures 4e, 5e, and 6e) showed reduced CCl_4_-induced injury, resulting in Level 1 steatosis (in Zones 1, 2, and 3), a moderate number of ballooned cells and inflammation, and an absence of necrotic or apoptotic cells (Figures 4e, 5e, and 6e). Conversely, the PSM + CCl_4_ rats (Figures 4f, 5f, and 6f) showed that PSM was a more effective pretreatment than silymarin, as the liver damage was less severe. The rats exhibited Level 1 steatosis, primarily in the porta structure (Figure 5f), a few ballooned cells (Figures 4f and 6f), less inflammation, and an absence of necrotic and apoptotic cells (Figures 4f and 6f).

## 4. Discussion

### 4.1. Immunomodulatory Activity of the PSMs

The spleen is the largest secondary lymphoid organ in the body and performs essential immunological functions along with its role in hematopoiesis and red blood cell elimination [31]. The PSM increased the in vitro splenocyte cellular metabolic activity (Figure 1a). However, the effect was lower than that of papache insoluble melanins at 25 *μ*g/mL [5]; the authors suggest the number of phenolic groups in the insoluble melanins contributes to such difference. This hypothesis is supported by the positive correlation between the immunomodulatory activity and the content of phenolics in mulberry, strawberry, and onion [25]. Furthermore, melanins in PSM are associated with carbohydrates, which could contribute to the immunomodulatory properties of PSM [23]. In this regard, polysaccharides of different sources are inducers of splenocyte proliferation: for example, *Crataegus pinnatifida* pollen (33% at 50 *μ*g/mL) [32], strawberry (36% at 250 *μ*g/mL), and mulberry leaves [33].

The in vivo data corroborated the PSM mitogenic activity, observing that splenocyte proliferation was higher in PSM-treated mice (Figure 1b). It has been proposed that plant melanins act as mitogens or superantigens, but the associated molecular pathways have not been described. However, suggested possible mechanisms include the induction of splenocyte proliferation through the TLR2/MyD88/JNK pathway by upregulating the expression of the B-cell activating factor (BAFF) and activating NF-*κ*B induced by B-cell expression [34]. Another possibility is based on findings with *Nigella sativa* melanins, which modulate cytokine levels by interacting with the toll-like receptor 4 (TLR4), leading to increased interleukin-1*β* (IL-1*β*) levels. IL-1*β* is an immunomodulatory cytokine secreted by activated monocytes through interaction with TLR2, its main receptor, and p38 mitogen-activated protein kinase (p38-MAPK) pathway [35]. The MAPK/ERK pathway promotes cell proliferation and reduces apoptosis [36]. Different vegetal melanins stimulate the splenic tissue: for example, mice treated with black tea melanins (50–200 mg/kg bw) showed the highest antibody response at 75 mg/kg bw [18]. Melanins also show different effects on the immune system. Mice treated with *Echinacea* melanins (10 mg/day) showed monocyte activation via TLR2 and increased IFN-*γ* levels in the spleen and IgA and IL-6 in PPs [17]. *Nigella sativa* melanins activate the NF-*κ*B signaling pathway and increase the mRNA levels of TNF-*α*, IL-6, and VEGF in monocyte and THP-1 macrophage cell lines [16, 37]; these melanins are suggested to treat immunomodulatory disorders [17]. Besides, melanins have been proposed as adjuvants with peptide vaccines used to treat cancer; this combination in mice favors the absorption in the lymph nodes [38]; thus, melanins could modulate the immune response by participating as a mitogen and in the immunologic cell activation. The in vitro and in vivo mitogenic activity of PSM was similar to LPS (positive control), which acts on B lymphocytes, demonstrating the PSM immunomodulatory potential. Further studies are needed to determine the type of lymphocytes and activation pathways affected by PSM in the spleen.

MLN and PP are part of the gut-associated lymphoid tissue (GALT), the largest lymphatic organ in the human body, contributing to more than 50% of the body's lymphocytes. The MLN comprises the highest lymph node concentration in the body and is in the mesentery base, whereas the PPs are lymph nodes distributed in the intestinal mucosa and submucosa. Consequently, in vivo evaluation of lymphocyte proliferation and activation in these lymphatic compartments is very important; MLN and PP are more exposed to antigens (e.g., commensal or pathogen microorganisms and alimentary components) than other lymphoid organs [39].

Mice treated with PSM showed T *γδ* lymphocyte activation (CD69^+^) in the MLN but suppression in the PP (Figure 3c). T *γδ* lymphocytes are a small population of primarily tissue-resident lymphocytes with innate and adaptive properties [40]. The activation of T *γδ* lymphocytes in MLN is interesting since the characteristics of these cells make them potential agents for cancer immunotherapy: cellular tropism, antitumor activity independent of neoantigen loading, and conventional presentation of MHC-dependent antigens, in addition to presenting a combination of typical functions of T cells and natural killer cells [41]. This finding agrees with one of the traditional uses of *Randia echinocarpa*. Although the antitumor functions of murine T *γδ* cells have been attributed to interferon-secreting (IFNc^+^) T *γδ* cells, recent studies have implicated IL-17 (IL-17^+^) producing T *γδ* cells in tumor growth and metastasis. However, the IL-17^+^ T *γδ* cells are rare in humans, and most studies have been focused on the protective role of T *γδ* cells against cancer [40].

On the other hand, it has been reported that the PP microenvironment favors a low T-cell reactivity to essential physiological stimuli required to start the immune response; this condition could be due to a higher IL-4 and IL-10 expressions in PP than in MLN [42, 43].

In contrast with the T *αβ*, the T *γδ* lymphocytes recognize ligands with high variation in size, composition, and molecular structure, including MHC and non-MHC cell surface molecules, soluble proteins, smaller peptides, phospholipids, and prenyl and sulfatide pyrophosphates. Several molecules are recognized in complexes with other molecules [44], a phenomenon that could happen with the PSM. The chemical characterization showed that PSMs are complexes of melanins with carbohydrates and lipids [23], probably involved in the T *γδ* lymphocyte activation.

The T *γδ* lymphocyte activation by dietary components has been scarcely studied. However, it is reported that white mistletoe increases IL-12 levels [45], a cytokine involved in the proliferation and cytotoxicity of T *γδ* lymphocytes. Moreover, white mistletoe extracts (50–500 mg/L) induce an in vitro concentration-response increment of T *γδ* cell proliferation [46]. On the other hand, in mice with an alimentary allergy and sensitized to ovalbumin, consumption of apple condensed tannins decreased the anaphylaxis severity, an activity that was associated with increased T *γδ* lymphocyte levels in the intestinal epithelium of mice fed with tannins [47]. Furthermore, the expression of T *γδ* lymphocytes in MLN and PP is increased in rats fed with dietary cocoa (10% w/w), suggesting this cell line participates in the intestinal immunological response in rats [26]. Consequently, the PSM could be a beneficial therapeutic agent because it can induce proliferation, suppression, and activation of immunocompetent cells, specifically, activating T *γδ* lymphocytes that show cytotoxic capacity independent of the MHC recognition.

### 4.2. Hepatoprotective Activity of the PSMs

The CCl_4_ metabolism by the enzymes CYP2E1, CYP2B, and CYP3 increases its toxicity, producing the highly unstable trichloromethyl radical that reacts with biomolecules (e.g., proteins, nucleic acids, and lipids) and affects the cellular integrity [48]. Similarly, an uncontrolled increase of reactive oxygen species (ROS) induces damage to membranes, DNA, and other molecules, contributing to developing pathologic conditions [49].

Oxidative stress induced by CCl_4_ and other agents results in hepatic damage characterized by increased levels of ALT, AST, ALP, and MDA, along with decreased levels of GSH. GSH is part of the antioxidant defense system in the body, inactivates the ROS, and prevents damage to membranes and organelles. On the other hand, MDA is a marker of lipidic peroxidation associated with physiopathologic oxidative stress processes [50]. In this context, PSM exhibited a hepatoprotective effect against the damage produced by CCl_4_ administration. PSM induced a better hepatoprotective effect against CCl_4_ toxicity than silymarin, an antioxidant and hepatoprotective compound reported in the literature as a control [27, 51, 52]. Compared with the ALT, ALP, AST, GSH, and MDA values in animals of the hepatotoxic group (AHTI), the prophylactic use of silymarin and PSM decreased the ALT levels, the ALP reduction was significant only for the PSM group, the GSH levels increased equally in both silymarin and PSM groups, and the MDA levels were lower in the PSM and silymarin groups and similar between them; whereas, contrary to the expected, these last two groups exhibited statistically similar AST values to the AHTI group (Table 1). These results align with previous findings on melanins' hepatoprotective effects against different agents. Melanins from various sources have exhibited hepatoprotective impact by improving the changes in the ALT, AST, GSH, and MDA markers. In murine models, tea melanins mitigate the effects of hydrazine [14, 19] and paracetamol exposure [53]. In a rat model, apricot kernel skin melanin reverses hydrazine-induced damage [28]. Fungal melanins from *Lachnum* spp. and *Auricularia auricula* counteract the ethanol-induced hepatic damage [20, 54, 55]. Furthermore, *A. auricula* melanins exhibited hepatoprotective effects against the lipopolysaccharide/d-galactosamine-induced damage in mice. Melanins from *Streptococcus parvus* protect against diethylnitrosamine-induced hepatic injury [21]. However, discrepancies related to hepatic damage parameters were noted in some studies. For instance, in mice treated with ethanol, AST levels showed a slight increase (7.8%), while the decreasing effect of *A. auricula* melanin on AST (4.5%–7.7%) was lower than that observed for the ALT levels (9.8%–32.9%) [54]. In rats damaged with CCl_4_, ALT levels were unaffected, and slight changes were registered in AST levels despite evident liver damage. The authors suggested that animals exhibited spontaneous recovery after discontinuing CCl_4_ administration [22]. In rats damaged by diethylnitrosamine, the hepatoprotective effect of *Streptococcus parvus* melanin was better at 10 mg/kg bw than at 20 mg/kg bw; at the latter dose, AST levels were statistically similar to those in animals treated only with diethylnitrosamine [21]. This study showed unexpected results in AST levels, where values among rats treated with silymarin + AHTI, PSM + AHTI, and AHTI were statistically similar, indicating little or no protection against the CCl_4_-induced hepatic injury (Table 1). However, other parameters and data in this study demonstrated the hepatoprotective effect of both silymarin and PSM. As observed in the studies mentioned above, variations in AST could provide insight into the result for the PSM + AHTI group. Furthermore, the elevated serum AST levels might originate from other organs, such as the heart and muscles [56].

Carbon tetrachloride exposure in animal organisms increases the synthesis of fatty acids and triacylglycerides from acetate. In rats treated with CCl_4_, the metabolism of CCl_4_ increases the acetate transport into liver cells, inhibits *β*-oxidation, and decreases the cellular VLDL mobilization [57]. Consequently, the availability and esterification of fatty acids intensify within liver cells, promoting vacuolar lipid accumulation in the hepatocyte cytoplasm [58], a phenomenon consistent with the observations of this study. In this regard, the silymarin pretreatment decreased the macro- and microvesicular steatosis in rats with AHTI (Figures 4, 5, and 6). This protection could be due to the flavonoids in silymarin (e.g., silybin, isosilybin, silydianin, and silychristin) that avoid the damage of hepatocyte membranes and the entry of toxic substances, increasing the protein synthesis and liver regeneration capacity [59].

The PSM had a better steatosis reduction than silymarin in rats with CCl_4_-induced AHTI (Figures 4, 5, and 6), demonstrating its hepatoprotective effect. The hepatoprotective potential of melanins finds further support in histopathological assessments on murine models. Specifically, studies involving melanins sourced from apricot kernel skin [28], *Lachnum* YM226 [20], *Nadoniella nigra* [60], *A. auricula* [54, 55, 61], *Inonotus obliquus* [22], and *S. parvus* [21] have provided evidence of their efficacy in safeguarding hepatic tissues.

Several studies with melanins support their hepatoprotective activity, which is associated with activating the enzymatic antioxidant system. For instance, the reduction in SOD activity induced by paracetamol in mice is counteracted by pretreatment with tea melanin (10–40 mg/kg bw) [53]. In murine hepatoprotection models, increased levels of key antioxidant enzymes such as SOD, GPx, and CAT were reported upon administration of melanins derived from different sources. These sources include apricot kernel skin [28], *Lachnum* YM226, and *L.* YM156 [20, 62], as well as *A. auricula* [54, 61]. Furthermore, in mice damaged with paracetamol, the GPx levels in animals pretreated with synthetic melanin nanoparticles equaled those of healthy mice [63].

Melanins have also improved the immune response in the hepatoprotective model. Mice treated with paracetamol exhibited a decrease in the count of B lymphocytes and levels of antibodies. However, pretreatment with tea melanins (30–40 mg/kg bw) prevented paracetamol-induced intoxication and elevated the levels of B lymphocytes [53]. Mice damaged with ethanol showed increased levels of IL-6, TNF-*α*, NF-*κ*B, MCP-1, COX-2, and iNOS, and the *Lachnum* YM226 melanin decreased the levels of these inflammatory parameters [20]. Furthermore, in mice with liver damage induced by lipopolysaccharide/d-galactosamine, treatment with *Lachnum* YM30 melanin reduces the NF-*κ*B levels, which probably contributes to the reduction of inflammation and liver injury [64]. A similar trend was observed in mice with hepatic damage induced by Cd; the administration of *Lachnum* YM156 melanin upregulated the expression of antioxidant genes such as Nrf2 and reduced the levels of TNF-*α*, IL-1*β*, NF-*κ*B p65, and iNOS, thereby enhancing the anti-inflammatory response [62]. In rats with obesity induced by monosodium glutamate, an elevation of proinflammatory cytokines IL-1*β* and IL-12B p40 was registered, coupled with downregulation of the anti-inflammatory system, as evidenced by reduced IL-4, IL-10, and TGF-*β* levels. Treatment with the *Nadsoniella nigra* melanin led to the activation of the anti-inflammatory system, as indicated by increased IL-10 and TGF-*β* levels and a decrease in IL-1*β* and IL-12B p40 [60]. In a model of ethanol-induced damage in mice, Nrf2 and other antioxidant genes were downregulated at the transcriptional and translational levels. However, pretreatment with *A. auricula* melanin reversed these changes [54]. Moreover, the administration of synthetic melanin nanoparticles blocked the overexpression of TNF-*α* and IL-6 induced by LPS in macrophages. This treatment also reduced the number of proinflammatory CD86^+^ macrophages in the liver of mice and the levels of proinflammatory cytokines in serum (TNF-*α*, IL-6, and IL-1B) [63]. In mice with paracetamol-induced damage, pretreatment with synthetic melanin nanoparticles decreased TNF-*α* and IL-6 levels in liver tissue. These findings suggest that PSM may exert its effects by modulating the immune response.

## 5. Conclusions

The soluble melanins of the *Randia echinocarpa* fruit exhibited immunomodulatory activity, characterized by enhanced splenocyte cellular metabolic activity in vitro and splenocyte proliferation in vivo. Additionally, the soluble melanins showed distinct modulation of T *γδ* lymphocytes in the MLNs and PPs, integral components of the GALT. Significantly, the hepatoprotective activity of the soluble melanins from *R. echinocarpa* surpassed that of silymarin, a known hepatoprotective agent. Given these properties, PSM may be considered a promising clinical alternative for liver disease prevention and an immunopotentiating agent for immunosuppressed patients, with potential applications as a nutraceutical, pharmaceutical formulation, or functional food ingredient.

## Figures and Tables

**Table 1:** Effect of papache soluble melanins (PSMs) (33 mg/kg bw) on levels of hepatic enzymes and malondialdehyde (MDA) in serum and reduced glutathione (GSH) in liver tissue of Wistar rats with acute hepatotoxic injury (AHTI).

**Group**	**ALT (U/L)**	**AST (U/L)**	**ALP (U/L)**	**GSH (ng/mg tissue)**	**MDA (nmol/mL serum)**
Control	15.03 ± 2.682^a^	37.14 ± 7.545^a^	247.42 ± 44.520^a^	189.61 ± 21.708^a^	4.35 ± 0.702^a^
Vehicle	10.61 ± 3.308^a^	31.69 ± 3.375^a^	262.35 ± 52.914^a^	179.75 ± 19.312^a^	4.57 ± 0.476^a^
PSM	23.65 ± 7.654^ac^	47.31 ± 18.377^a^	300.70 ± 31.236^a^	165.41 ± 46.701^a^	5.42 ± 0.660^a^
PSM + AHTI	64.02 ± 24.253^bc^	318.62 ± 77.642^b^	355.47 ± 67.093^ab^	252.15 ± 34.747^b^	7.85 ± 0.783^b^
Silymarin + AHTI	138.53 ± 1.235^b^	274.85 ± 52.359^b^	405.31 ± 39.676^bc^	266.49 ± 10.757^b^	8.12 ± 0.869^b^
AHTI	218.85 ± 6.502^d^	225.92 ± 83.957^b^	453.37 ± 92.204^c^	127.96 ± 70.422^a^	10.25 ± 0.976^c^

*Note:* Results are the mean ± standard deviation (*n* = 6; ALT, AST, and ALP) (*n* = 3, GSH and MDA). Different letters in the same column indicate a significant difference between groups (*p* < 0.05). Treatments: control, pure water ad libitum; vehicle, saline solution (0.09% w/v); PSM (33 mg/kg bw via i.p.); AHTI (CCl_4_ 2 mL/kg bw via i.p.); silymarin + AHTI (silymarin 50 mg/kg bw via p.o. and CCl_4_ 2 mL/kg bw via i.p.); PSM + AHTI (PSM 33 mg/kg bw via i.p. and CCl_4_ 2 mL/kg bw via i.p.).

Abbreviations: ALP, alkaline phosphatase; ALT, alanine aminotransferase; AST, aspartate aminotransferase; GSH, reduced glutathione; MDA, malondialdehyde.

## Data Availability

Data are available on request from the authors.

## Nomenclature

AHTI: acute hepatotoxic injury
ALP: alkaline phosphatase
ALT: alanine aminotransferase
AST: aspartate aminotransferase
CAT: catalase
GPx: glutathione peroxidase
GSH: glutathione
B: lymphocyte B
LPS: lipopolysaccharide
T: lymphocyte T
MDA: malondialdehyde
MLNs: mesenteric lymph nodes
NASH CRN: Nonalcoholic Steatohepatitis Clinical Research Network
PPs: Peyer's patches
PSMs: papache soluble melanins
SOD: superoxide dismutase
